# Sperm selection by birefringence: a promising non-invasive tool to improve ICSI outcomes

**DOI:** 10.5935/1518-0557.20220055

**Published:** 2023

**Authors:** Mariana Antunes Ribeiro, Michele Gomes Da Broi, Mauro Bibancos de Rose, Andrea Garolla, Carlo Foresta, Aristides Manoel dos Santos Bragheto, Daniel Gustavo Faúndes Hardy

**Affiliations:** 1 Human Reproduction Institute - Fivmed, Campinas, São Paulo, CEP 13073-002, Brazil; 2 Section of Clinical Pathology and Unit for Human Reproduction Pathology, Department of Medicine, University of Padova, Padova, Italy

**Keywords:** sperm DNA fragmentation, birefringence, ICSI, clinical pregnancy

## Abstract

**Objective:**

Despite higher sperm DNA fragmentation may affect intracytoplasmic sperm injection (ICSI) outcomes, sperm selection protocols do not evaluate this parameter. Therefore, sperm’s head birefringence has been suggested as an adjuvant of seminal processing to select viable sperm for couples with severe male factor. Considering men with normal seminal parameters may also curse with DNA fragmentation, the aim of this study was to evaluate the impact of sperm selection by birefringence on ICSI outcomes in couples with different infertility factors compared to those submitted to conventional sperm selection.

**Methods:**

In this case-control study, medical records from 181 couples who underwent ICSI from January 2018 to August 2020 (107 from the Conventional and 74 from the Birefringence group) were included in the study. Clinical characteristics and ICSI outcomes were compared between the groups using Student’s t test or Chi-square test (*p*<0.05) and a multivariate logistic regression model was applied regarding clinical pregnancy.

**Results:**

Despite the Birefringence group showed higher female age (*p*=0.01), lower seminal sperm concentration (*p*<0.01) and higher sperm DNA fragmentation (*p*<0.01), those patients cursed with both higher cleavage rate (*p*=0.04), clinical pregnancy rate per transfer (*p*=0.03) and clinical pregnancy rate per initiated cycle (*p*=0.02). The logistic regression showed a positive group effect on clinical pregnancy.

**Conclusions:**

The findings suggest a positive clinical impact of this cheap and easily reproducible adjuvant technique on ICSI outcomes in couples with different infertility factors. If confirmed by further methodologically appropriate studies, the sperm’s head birefringence could be considered to improve the reproductive chances of those patients.

## INTRODUCTION

Originally developed to treat couples with severe male infertility factor (Palermo *et al*., 1992), intracytoplasmic sperm injection (ICSI) has been widely extended for other infertility conditions treatment (O’Neill *et al*., 2018). However, although the introduction of ICSI has reduced the minimum seminal parameters required for Assisted Reproduction Treatments (ART), the outcomes obtained are still below expectations (Adamson *et al.*, 2018; Ishihara *et al*., 2015). It is known that the quality of seminal samples is strictly related to the predisposition to chromosomal errors and to the incidence of abnormal sperm (Bernardini *et al*., 2000; Gianaroli *et al.*, 2005). Moreover, infertile men have a higher frequency of gametes with altered DNA than fertile men and the frequency of anomalies increases proportionally to the severity of the male factor (Bernardini *et al*., 2000; 2004; 2005; Braham *et al.*, 2019; Calogero *et al*., 2003; Esquerré-Lamare *et al*., 2018; Gianaroli *et al*., 2005; Kodama *et al*., 1997; Sakkas *et al.*, 2003; Simon *et al.*, 2017; Zini *et al.,* 2001). In addition, even infertile men with normal seminal parameters may also show high DNA fragmentation (Kodama *et al*., 1997; Zini *et al*., 2001). Seminal processing methods used to select the spermatozoa to be injected into oocytes allow the selection of spermatozoa considering their motility and morphology (Boomsma *et al*., 2019; Lepine *et al*., 2019). However, as these procedures do not use molecular criteria for sperm evaluation, the functional sperm analysis is compromised, which could negatively affectART results (Lepine *et al*., 2019; Lewis, 2007). The main issue is not only having a mobile and morphologically normal sperm, but also having the ability to fertilize the oocyte and generate viable embryos.

Sperm DNA fragmentation (sDNAfrag) has been the subject of several studies on infertility. Sperm DNA is highly compacted in the form of chromatin through specific histones called protamines that keep DNA protected (Gill *et al*., 2019; Santi *et al*., 2018; Tanaka *et al*., 2022). Unfortunately, as in ICSI there is no selection based on DNA integrity, it is possible that functionally abnormal spermatozoa are injected. Spermatozoa with fragmented DNA are known to be associated with lower fertilization rates (Ozmen *et al*., 2007), embryonic development arrest, low implantation and pregnancy rates (Benchaib *et al*., 2007; Virro *et al*., 2004), increased incidence of spontaneous abortion (Dar *et al*., 2013), lower rates of live births (Simon *et al*., 2013) and/or high morbidity in offspring originated by ICSI (Fernández-Gonzalez *et al*., 2008). Thus, the sperm DNA integrity may be considered of extreme importance for obtaining a pregnancy (Agarwal & Allamaneni, 2005). Therefore, the analysis of sDNAfrag may be an important tool associated with conventional seminal processing methods to select viable sperm with reproductive potential and, thus, obtain better ICSI results (Zhao *et al*., 2014).

Nevertheless, most of the sDNAfrag tests preclude the reproductive use of the analyzed cells (Sharma *et al*., 2021). In this sense, sperm birefringence evaluation has been considered as a non-invasive and low-cost methodology that allows the selection of sperm with intact DNA (Garolla *et al*., 2014; Ghosh *et al*., 2012; Gianaroli *et al*., 2008; 2010). It is based on the decomposition of a ray of light into two beams as it passes through an anisotropic material under a polarized light microscope (Baccetti, 2004; Gianaroli *et al*., 2010). Accordingly, birefringent sperm head indicates viable cell while the absence of birefringence indicates sDNAfrag (Garolla *et al*., 2014). In this context, preliminary data suggested that, in patients with severe male factor infertility, sperm selection by birefringence might distinguish spermatozoa with greater reproductive potential and embryonic viability, without affecting their vitality or motility (Ghosh *et al*., 2012; Gianaroli *et al*., 2008; 2010). Authors also suggested that, in normozoospermic men, combining birefringence and motile sperm organelle morphology examination under high magnification (MSOME) methods using a single microscope could increase the likelihood of selecting sperm with intact DNA (Garolla *et al*., 2014).

It is known that conventional ICSI procedures do not select spermatozoa with intact DNA, even though men with normal seminal parameters may also evidence sperm with high DNA fragmentation, which could impair their reproductive success. Although there is evidence that the injection of birefringent sperm may improve ICSI outcomes in couples with severe male factor infertility when compared to nonbirefringent sperm, it is still unknown whether performing or not birefringence interferes on ART outcomes when applied to couples with distinct infertility factors. Thus, the aim of this study was to evaluate the impact of sperm selection by birefringence on ICSI outcomes in couples with different infertility factors compared to conventional sperm selection.

## MATERIALS AND METHODS

### Study design, Settings and Ethics

In this retrospective observational case-control study, medical records of couples undergoing Assisted Reproduction Treatments from January 2018 to August 2020, were assessed for eligibility, and those who met the selection criteria were included in the study.

The study was approved by the Research Ethics Committee designated (Investiga - Research Institutes; Process number 29697420.4.0000.5599).

### Participants/Eligibility Criteria

Couples whose women was under 37 years old, who have undergone ICSI using their own oocytes, using semen samples obtained from the ejaculate and freshly processed were considered eligible.

Infertility factors were identified based on couple anamnesis, imaging exams, symptoms and clinical history. Specifically, patients were diagnosed as poor ovarian responders based on Bologna Criteria (Ferraretti *et al.*, 2011). Endometriosis was diagnosed based on clinical history and imaging exams.

Patients who had more than three previous unsuccessful cycles, who used cryopreserved gametes or who performed pre-implantation genetic testing on embryos (embryo biopsy) were excluded.

The patients were stratified into the groups without birefringence (Conventional) or with birefringence (Birefringence) according to the sperm selection by the analysis of conventional morphology and motility alone or associated to sperm birefringence, respectively. The criterion for the realization or not of this methodology was the couple’s agreement to add it to the treatment since it was offered to all patients in the service.

### Assisted Reproduction Procedures

#### Stimulation Protocol

Pituitary blockage was performed with the administration of a GnRH antagonist and the ovarian stimulation was started on the 3^rd^ day of the menstrual cycle with the administration of recombinant FSH (FSHr: Gonal-F^®^, Serono, Brazil; Puregon^®^, Organon, Brazil), 150 to 300 IU per day, for 6 days. Follicular development was monitored daily by transvaginal ultrasound (USTV) from the 7^th^ day of stimulation and the dose of FSHr was adjusted according to follicular growth and maintained until the day of the trigger. Trigger for final follicular maturation was done when at least 3 follicles reached a size larger than 17 mm, with 0.2 mg triptorelin (Gonapeptildaily 0,1mg/ml Ferring GmbH Wittland 11 - D-24109 - Kiel, Germany) or 0.2 mg leuprorelin (Lupron kit 5mg/ml, Famar L’Aigle Saint-Remy-Sur-Avre - France).

#### Oocyte retrieval, denudation, Intracytoplasmic sperm injection and birefringence

Oocytes were obtained 35 hours later from patients under general intravenous anesthesia with propofol (Diprivan, Astra-Zeneca, Brazil) and fentanyl (Fentanil, Janssen-Cilag, Brazil). Follicles were aspirated via endovaginal route guided by a transvaginal ultrasound transducer using a standard single-lumen needle (CDD Laboratory, France) with a constant artificial aspiration pressure of 100 mmHg.

To identify and isolate the cumulus-oocyte complexes (COC), the aspirated material was transferred to Petri dishes, previously heated to 37°C, without culture medium. Once identified, the COCs were isolated from the follicular fluid and placed on a separate plate. COCs were washed in Multipurpose Handling Medium - Complete culture medium (MHM-CF, Irvine Scientific, USA) to remove blood and cell debris and were cultured in Continuous Single Culture Complete with HSA (CSCM-C) medium (Irvine Scientific ) at 6.0% CO_2_, 5.0% O_2_ and 37°C. Two hours after oocyte retrieval, the oocytes were stripped and evaluated for their maturity stage.

All semen samples were processed by density gradient centrifugation.

All metaphase II (MII) oocytes were inseminated by intracytoplasmic sperm injection (ICSI) 3 to 5 hours after oocyte retrieval. In conventional ICSI, sperm were selected according to their morphology, and evaluated under an inverted microscope (Ti Eclipse; Nikon) for normal morphology and motility.

In ICSI with birefringence, sperm were evaluated in a light polarized microscope (Ti Eclipse; Nikon) equipped with high-power differential interference contrast optics and with Hoffman contrast (40X objective) and polarizing lenses.

The analysis of birefringence distinguished between reacted and nonreacted spermatozoa, and those whose head reacted (light refraction) were selected, suggesting that there was no DNA fragmentation (Garolla *et al*., 2014).

Fertilization was evaluated approximately 16 to 18 hours after ICSI. The presence of two pronuclei and two polar bodies was considered as normal fertilization. Abnormal fertilization was the presence of one, 3 or more pronuclei after ICSI.

Embryonic quality was analyzed approximately 67-69 hours after ICSI (D3), based on the number and symmetry of the blastomeres, percentage of fragmentation and presence or absence of multinucleation (Alpha Scientists in Reproductive Medicine & ESHRE Special Interest Group of Embryology, 2011). The embryonic quality was again analyzed 114-118 hours (and, in specific cases, 138-142 hours and 162-166 hours) after ICSI, in the blastocyst stage, considering the morphology of the internal cell mass (MCI) and the trophoectoderm. Blastocysts fully expanded and hatched, with an easily discernible MCI, composed of many compacted cells, firmly adhered to each other and with a trophectoderm with many cells forming a cohesive epithelium were considered to be of high quality (HQ) ( Alpha Scientists in Reproductive Medicine & ESHRE Special Interest Group of Embryology, 2011).

As a routine of the service, all freshly produced embryos were cryopreserved (freeze all method) by the vitrification technique, following the commercial kit Irvine protocol (Irvine Scientific, California). Embryo transfer was performed in a later cycle, after endometrial preparation.

#### Endometrial preparation

The endometrial preparation started on the first day of menstruation of the embryo transfer cycle, with the performance of USTV and administration of 2 mg of estradiol valerate (Primogyna, Bayer SA, Brazil) twice a day, for 10 days. On the 11^th^ day, a new USTV was performed and, if the endometrium was trilaminate, with thickness >7 mm, supplementation with 400 mg of progesterone (Utrogestan^®^, Besins Healthcare, Brazil) was started, twice a day for 5 days, when embryo transfer was performed. Progesterone supplementation was maintained until the β-hCG test was performed, 9 days after the transfer. In case of a negative test, the treatment stopped. In case of pregnancy, treatment was continued until the 12^th^ week of pregnancy.

#### Variables

The primary endpoint of this study was clinical pregnancy. The following parameters were assessed: female age, BMI, associated infertility factor, seminal parameters of the partner, total dose of FSH, number of oocytes retrieved, number of mature oocytes, number of injected oocytes, number of fertilized oocytes, fertilization rate, number of cleaved embryos, cleavage rate, number of blastocysts formed, blastulation rate, number of high quality (HQ) embryos formed, number of transferred embryos, number of HQ embryos transferred, implantation rate, biochemical pregnancy rate, and clinical pregnancy rate *per* transfer and *per* initiated cycle were evaluated.

The fertilization rate was defined as the ratio between the number of oocytes with two pronuclei and two polar bodies to the number of injected oocytes x 100. The cleavage rate was based on the number of cleaved embryos divided by the number of fertilized oocytes x 100. The blastulation rate was obtained from the ratio between the number of blastocysts formed by the number of cleaved embryos x 100. The biochemical pregnancy rate consisted of the number of patients with a positive β-hCG result divided by the number of transferred patients x 100. A clinical pregnancy was defined by the presence of a gestational sac and fetal heartbeat on the ultrasound (US). The clinical pregnancy rate *per* transfer was calculated by the ratio between the number of patients with gestational sac and embryo heartbeat on the US and the number of transferred patients x 100. The clinical pregnancy rate *per* initiated cycle was calculated by the ratio between the number of patients with gestational sac and embryo heartbeat on the US and the number of cycles x 100. The implantation rate was expressed by the ratio between the number of gestational sacs seen on US and the number of transferred embryos x 100.

### Potential sources of bias

Selection bias was avoided by evaluating medical records from all patients who underwent ovarian Assisted Reproduction Treatments in the period of the study.

Reporting bias was avoided by presenting and analyzing all results obtained.

### Sample size

The sample consisted of all patients considered eligible according to the eligibility criteria of the study and who underwent ovarian stimulation for ICSI in the period from January 2018 to August 2020.

### Statistical analysis

An exploratory analysis of the data was performed using measures of central position and dispersion and box-plot graphs.

A univariate analysis was performed, using Student’s *t* test, to compare quantitative variables between the groups. The Chi-square test was used to compare qualitative variables between the groups. A multivariate logistic regression model was applied to verify the covariables (female age, endometriosis factor infertility, male factor infertility and group) possibly associated with the endpoint clinical pregnancy.

Statistical analysis was performed using the SAS® 9.4 program. The level of significance adopted was 5%.

## RESULTS

### Flowchart

**Figure 1:**
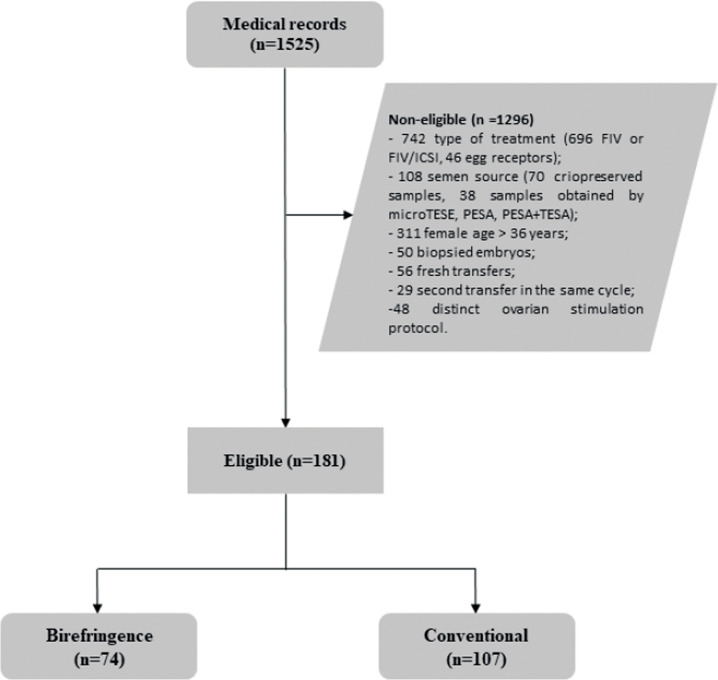
Flowchart of the patients selection.

Medical records of 1525 couples who underwent Assisted Reproduction Treatments from January 2018 to August 2020, were assessed for eligibility. Of them, 1296 were not considered eligible [742 because of the type of procedure (696 FIV or FIV/ICSI and 46 oocyte recipients)]; 108 in consequence of the sperm source (70 cryopreserved samples, 38 samples obtained by microTESE, PESA or PESA + TESA); 311 due to female age > 36 years; 50 because performed pre-implantation genetic testing; 56 in consequence of fresh embryo transfer; 29 because of subsequent transfers of the same stimulation cycle, 48 because of distinct ovarian stimulation protocols]. Thus, 181 patients were included in the study, of which 74 were stratified into the Birefringence group and 107 into the Conventional group (Figure 1).

### Clinical characteristics

Clinical characteristics of the groups are reported in Table 1. The Birefringence group showed higher female age (*p*=0.01), lower seminal sperm concentration (*p*<0.01) and higher sDNAfrag (*p*<0.01) than the Conventional group. The groups also showed differences regarding the infertility factors (*p*<0.01).

**Table 1:** Clinical characteristics and stimulation parameters of the Birefringence and Conventional groups.

Variables	Birefringence (n=74)	Conventional (n=107)	*p*
**Female age (years)**	32.81±2.61	31.75±3.13	0.01
**Infertility Factor** **Poor ovarian reserve** **Male factor** **Endometriosis** **Anatomic Disorder**	5.63% 80.28% 11.27% 2.82%	12.22% 48.89% 24.44% 12.82%	<0.01
**Semen parameters** **Concentration/mL** **Volume** **Progressive motility** **DNA fragmentation (%)**	14.80±14.84 2.71±1.51 49.71±18.21 37.17±14.09	23.62±20.38 2.73±1.33 50.09±20.53 16.95±10.78	<0.01 0.93 0.47 <0.01
**Trigger** **hCG** **GnRHa** **Mixed hCG and GnRHa**	74.07% 11.11% 14.81%	85.87% 6.52% 7.61%	0.20
**Total FSH dose**	1545.59±1027.45	1495.13±871.06	0.93
**Number of retrieved oocytes**	14.51±7.94	17.15±11.05	0.06
**Number of mature oocytes**	10.08±5.91	10.49±6.91	0.68

BMI: Body Mass Index. Data compared using *t* test or Chi-square test. Significance: *p*<0.05.

### ICSI outcomes

Regarding ICSI outcomes, the Birefringence group showed higher embryo cleavage rate (*p*=0.04), clinical pregnancy rate *per* transfer (*p*=0.03) and clinical pregnancy rate *per* initiated cycle (*p*=0.02) compared to the Conventional group (Table 2). Other ICSI outcomes were similar between the groups (Table 2).

**Table 2:** ICSI outcomes of the Birefringence and Conventional groups.

Variables	Birefringence (n=74)	Conventional (n=107)	*p*
Number of inseminated oocytes	9.43±5.27	9.51±5.58	0.92
Number of fertilized oocytes	7.74±4.79	7.85±4.83	0.87
Fertilization rate	80.85±19.69	83.13±17.44	0.41
Number of cleaved embryos	7.56±4.40	7.38±4.68	0.79
Cleavage rate	94.41±15.64	88.89±20.84	0.04
Number of blastocysts formed	4.97±3.53	5.02±3.67	0.93
Blastulation rate	64.82±26.39	65.60±28.99	0.85
Number of HQ blastocysts	2.43±2.26	2.51±2.67	0.84
Number of blastocysts transferred	1.54±0.50	1.67±0.47	0.09
Number of HQ blastocysts transferred	0.85±0.69	0.78±0.73	0.59
Biochemical pregnancy rate	47/67 (70.15%)	56/93 (60.22%)	0.19
Clinical pregnancy rate per transfer	45/67 (67.16%)	47/93 (50.54%)	0.03
Clinical pregnancy rate per initiated cycle	45/74 (60.81%)	47/107 (43.93%)	0.02
Implantation rate	56/103 (54.37%)	66/155 (42.58%)	0.06

HQ: High quality. Data compared using *t* test or Chi-square test. Significance: *p<*0.05.

The regression analysis including female age, endometriosis factor infertility, male factor infertility and group demonstrated only a group effect. In this sense, the birefringence showed a protective effect, increasing in 2.3 the odds to achieve clinical pregnancy (CI 95% 1.10 - 4.79; Table 3).

**Table 3:** Multivariate logistic regression to verify the factors associated with the endpoint clinical pregnancy.

Factor	ODDS RATIO	CI 95%	
**Female age**	0.915	0.807	1.039
**Endometriosis Factor Infertility**	0.974	0.393	2.410
**Male Factor Infertility**	1.376	0.574	3.297
**Group (Birefringence *vs*. Conventional)**	2.305	1.108	4.795

CI: Confidence Interval.

## DISCUSSION

Sperm DNA fragmentation has been considered as an important factor that may negatively affect ICSI outcomes (Benchaib *et al*., 2007; Dar *et al*., 2013; Ozmen *et al*., 2007; Simon *et al*., 2013; Virro *et al.*, 2004). However, the widely utilized methods for analyzing sDNAfrag preclude using the evaluated sperm in ART (Sharma *et al*., 2021). In this sense, sperm head birefringence has been proposed as a non-invasive tool to analyze sDNAfrag, enabling the subsequent sperm use in ICSI procedures (Garolla *et al.*, 2014; Ghosh *et al*., 2012; Gianaroli *et al*., 2008; 2010). Preliminary studies have shown that birefringence has a positive impact when applied for sperm selection in patients with severe male factor (Ghosh *et al*., 2012; Gianaroli *et al*., 2008; 2010). However, it is known that even men with normal seminal parameters may have high fragmented sperm DNA (Kodama *et al*., 1997; Zini *et al*., 2001), which could impair ICSI outcomes, especially those couples with no sDNAfrag suspected. There is evidence that the injection of birefringent sperm may improve ICSI outcomes in couples with severe male factor infertility when compared to nonbirefringent sperm, however, whether performing or not birefringence selection interferes with ART outcomes when applied to couples with distinct infertility factors still needs investigation. Thus, this study aimed to evaluate the impact of birefringence in selecting the sperm to be injected for ICSI procedure in couples with different infertility factors.

According to the present findings, despite showing older female age and higher sDNAfrag, the group of patients submitted to sperm selection using birefringence obtained higher rates of clinical pregnancy, both *per* transfer (67.16%) and *per* initiated cycle (60.81%), than the Conventional group (50.54%, 43.93%, respectively). In addition, the factor group was significantly identified as the only one impacting clinical pregnancy, suggesting that performing birefringence could be a protective factor for obtaining clinical pregnancy. The differences observed related to the group that did not undergo birefringence were approximately 16% in each significantly different rate, representing an expressive clinically relevant increase to pregnancy success. If this finding is confirmed in further studies with larger sample size, this may justify the higher clinical pregnancy rates found. In this sense, well-selected sperm could give rise to higher quality embryos with greater implantation potential, a hypothesis that still needs to be proven.

Our data corroborate positive results from previous studies with severe male factor infertility. Gianaroli *et al*. (2008) reported that sperm selection by birefringence resulted in higher percentages of good quality embryos on the third day and higher implantation rates in ICSI cycles compared to conventional sperm selection for men with severe male factor. Next, despite focusing in birefringence for acrosome reaction examination, the same group evidenced that in patients with oligoastenoteratospermia or azoospermia, the use of birefringent sperm selected for ICSI (including sperm obtained by testicular extraction) was associated with higher rates of implantation, clinical pregnancy and live births compared to nonbirefringent sperm (Gianaroli *et al*., 2010). There is also a report evidencing higher pregnancy rates and good quality embryos with the application of this technique in men with complete asthenozoospermia (Ghosh *et al*., 2012). Altogether those findings and our data suggest birefringence as a positive criterion for sperm selection in order to improve ICSI outcomes. Considering normal seminal parameters, Garolla *et al*. (2014) suggested that, in normozoospermic men, combining birefringence and MSOME methods using a single microscope could increase the likelihood of selecting sperm with intact DNA, although ICSI outcomes were not evaluated. However, associating birefringence to MSOME technology would difficult its application to all patients, since MSOME is a method that requires greater investment, more execution time, which may impact the laboratory’s routine. Given the advantages of birefringence, such as the easy and low-cost technology, which requires only team training and once used, it becomes fast and does not interfere with laboratory routine and considering it may increase in 16% the clinical pregnancy rates, birefringence alone seems to be a promising tool for sperm selection in ICSI cycles.

However, despite the encouraging results, it should be noted that this study has some limitations. The heterogeneous inclusion of infertility factors between the groups might represent a bias, since patients without birefringence had more infertility associated to the presence of endometriosis while the group with birefringence had more infertility associated to male factor. However, in logistic regression analysis, none of those factors was shown to interfere with clinical pregnancy, which reinforces the impact of the technique on the results. In addition, the endpoint here analyzed was clinical pregnancy, while live birth would better reflect ICSI success. Nevertheless, the sample size did not allow this analysis. Future studies with a greater casuistry will clarify this question.

The data presented here are preliminary but very promising, since birefringence seems to have a positive clinical impact on ICSI outcomes, representing a 16% increase in clinical pregnancy rate *per* transfer and *per* initiated cycle. Because it is a cheap and easily reproducible technique, it could be associated to ICSI treatments to improve the reproductive outcomes of those patients. Further studies are necessary to elucidate the impact of birefringence applied to distinct infertility factors on live birth rates.
